# Supplementary material for: The Shigella E3 ubiquitin ligase IpaH7.8 reprograms host kinase signaling to suppress NOX2-dependent oxidative burst responses in human monocytes
Source: bioRxiv. 2026 Apr 29:2026.04.28.721327. Preprint. [Version 1] doi: 10.64898/2026.04.28.721327 (PMC13142472; doi:10.64898/2026.04.28.721327)
Supplement: Supplement 1 [file NIHPP2026.04.28.721327v1-supplement-1.pdf]

bioRxiv preprint doi: https://doi.org/10.64898/2026.04.28.721327; this version posted May 1, 2026. The copyright holder for this preprint (which was not certified by peer review) is the author/funder, who has granted bioRxiv a license to display the preprint in perpetuity. It is made available under aCC-BY 4.0 International license.

Supplemental Figures

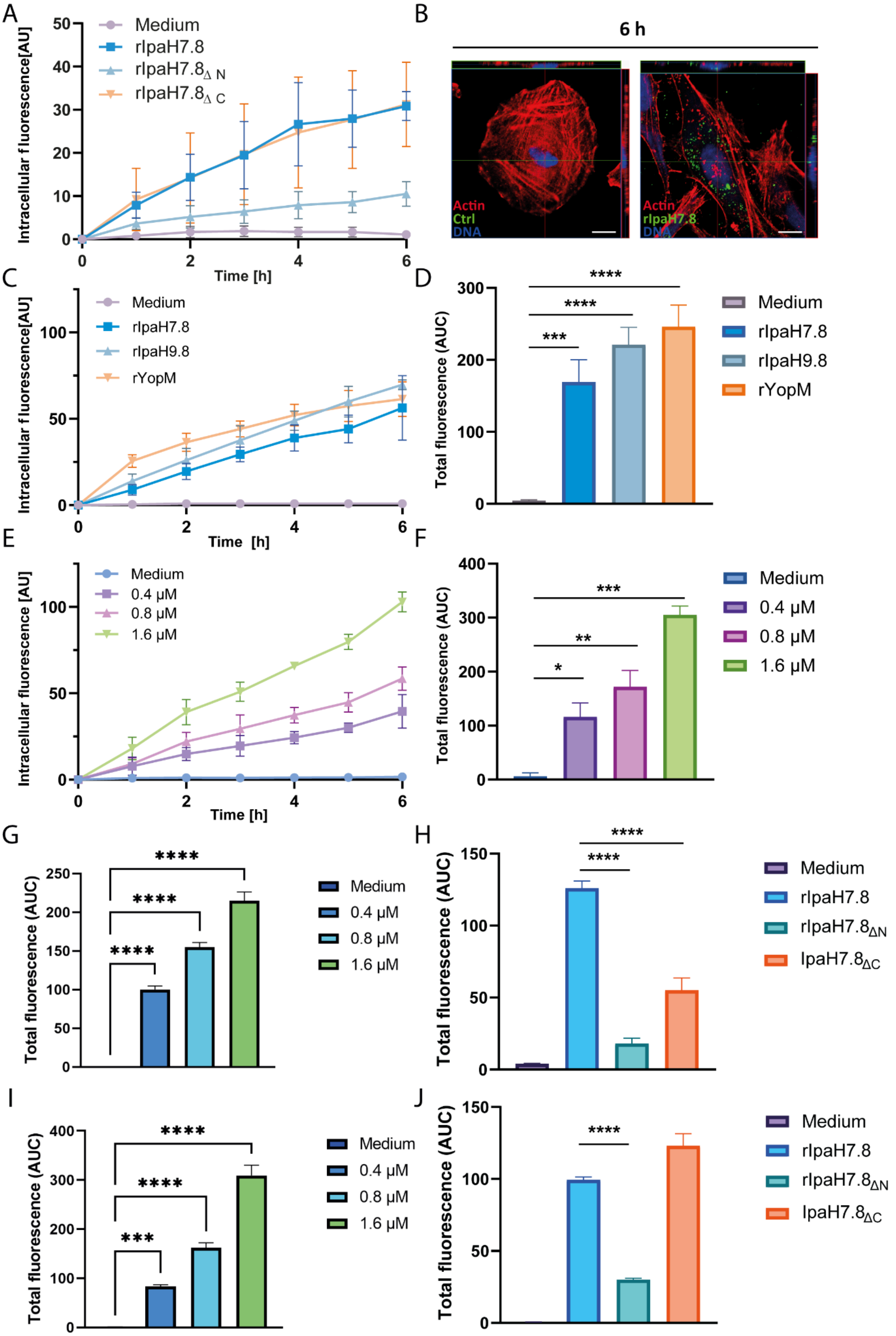

bioRxiv preprint doi: https://doi.org/10.64898/2026.04.28.721327; this version posted May 1, 2026. The copyright holder for this preprint (which was not certified by peer review) is the author/funder, who has granted bioRxiv a license to display the preprint in perpetuity. It is made available under aCC-BY 4.0 International license.

Supplemental Figure S1. T3SS-independent uptake of rIpaH7.8 in epithelial and immune cell lines. Flow cytometry-based quantification of intracellular fluorescence in HeLa (A, C-F), HEK293 (G, H) and THP-1 macrophages (I, J) incubated with increasing concentrations (0.4-1.6 µM) of FITC-labeled rIpaH7.8 (E, F, G, I) and rIpaH7.8/rYopM (C, D) or with 0.4 µM FITC-labeled rIpaH7.8ΔN or rIpaH7.8ΔC for up to 6 h (A, C, H, J). Medium-only samples served as controls. Data are shown as mean ± SD of three independent experiments; AU, arbitrary units. Area under the curve (AUC) values are shown for visualization. Statistical analysis by one-way ANOVA with Tukey's post hoc test; ns: not significant, *$p < 0.05$, **$p < 0.01$, ***$p < 0.001$, ****$p < 0.0001$ vs. medium. (B) Representative fluorescence microscopy images of HeLa cells incubated with 0.8 µM FITC-labeled rIpaH7.8 for 6 h. Actin (red), FITC-rIpaH7.8 (green), nuclei (blue). Scale bar, 10 µm. Images show merged channels from a single optical section.

bioRxiv preprint doi: https://doi.org/10.64898/2026.04.28.721327; this version posted May 1, 2026. The copyright holder for this preprint (which was not certified by peer review) is the author/funder, who has granted bioRxiv a license to display the preprint in perpetuity. It is made available under aCC-BY 4.0 International license.

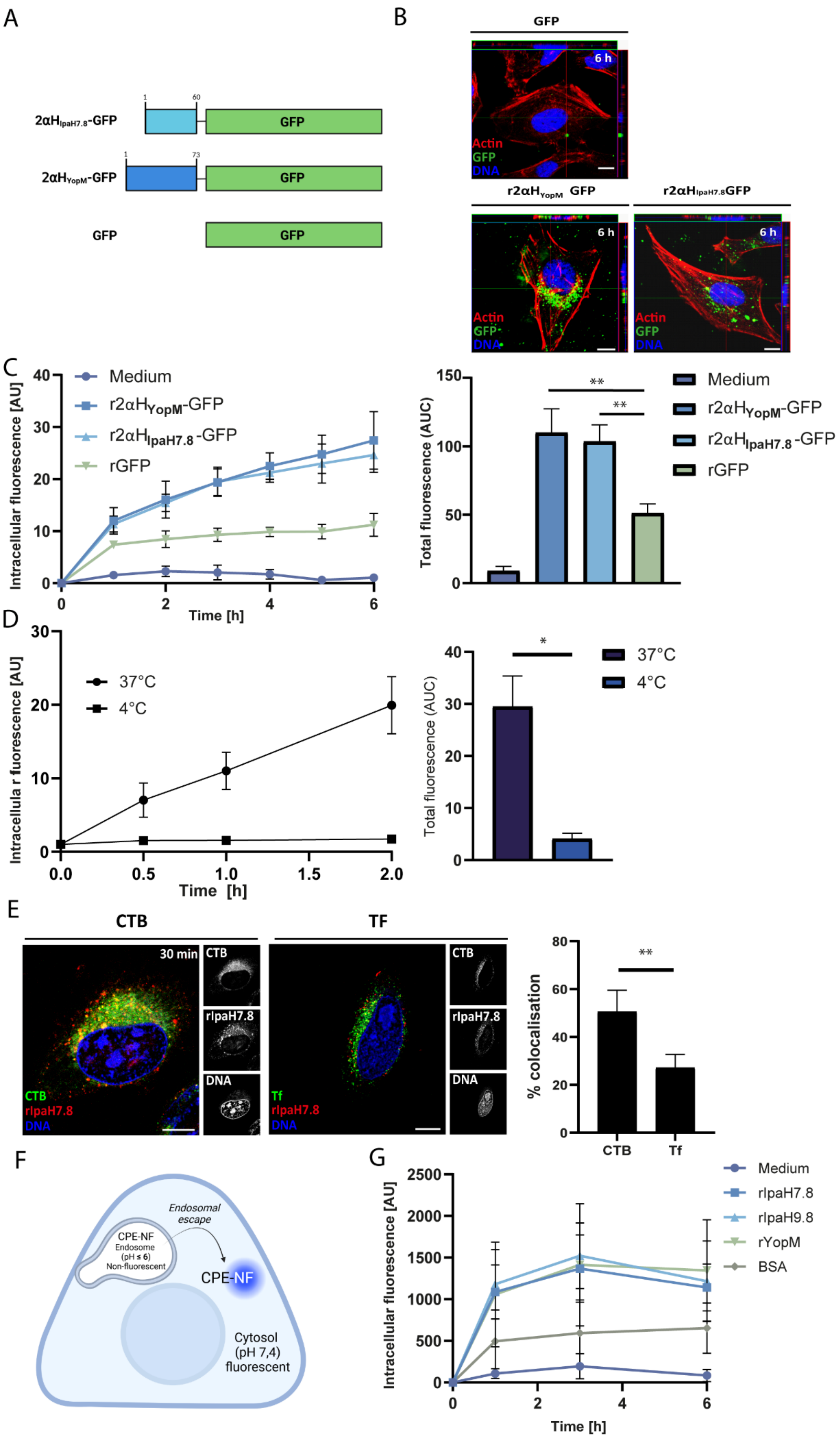

bioRxiv preprint doi: https://doi.org/10.64898/2026.04.28.721327; this version posted May 1, 2026. The copyright holder for this preprint (which was not certified by peer review) is the author/funder, who has granted bioRxiv a license to display the preprint in perpetuity. It is made available under aCC-BY 4.0 International license.

Supplemental Figure S2. Uptake and cytosolic delivery of IpaH7.8 GFP fusion constructs. (A) Schematic overview of GFP fusion constructs: 2αHIpaH7.8-GFP and 2αHYopM-GFP, containing the N-terminal domains of IpaH7.8 or YopM; rGFP served as control. (B) Representative fluorescence microscopy images of HeLa cells incubated with 0.8 µM FITC-labeled rGFP, 2αHYopM-GFP, or r2αHIpaH7.8-GFP for 6 h. Actin (red), protein (green), nuclei (blue). Scale bar, 10 µm. (C) Flow cytometry quantification of intracellular GFP fluorescence after incubation with 0.4 µM FITC-labeled proteins. Data show mean ± SD (n = 3); AUC values are indicated. One-way ANOVA with Tukey's post hoc test (ns, *p < 0.05 vs. rGFP). (D) Temperature-dependent uptake of Alexa Fluor 488-labeled rIpaH7.8 at 37 °C or 4 °C for up to 3 h (AUC; n = 3; paired t-test, *p < 0.01). Co-localization of Cy3-rIpaH7.8 with Cholera Toxin B-subunit CTB-FITC (lipid rafts) or transferrin-Alexa Fluor 488 (Tf) after 30 min. Quantification from six images are shown (paired t-test, **p < 0.01). Scale bar, 10 µm. (F) Experimental scheme for detecting cytosolic localization using the pH-sensitive dye 5(6)-carboxynaphthofluorescein (NF). (G) Flow cytometry of HeLa cells incubated with 0.4 µM NF-labeled rIpaH7.8, rIpaH9.8, rYopM, or BSA for up to 6 h. Data show mean ± SD (n = 3); AU, arbitrary units.

bioRxiv preprint doi: https://doi.org/10.64898/2026.04.28.721327; this version posted May 1, 2026. The copyright holder for this preprint (which was not certified by peer review) is the author/funder, who has granted bioRxiv a license to display the preprint in perpetuity. It is made available under aCC-BY 4.0 International license.

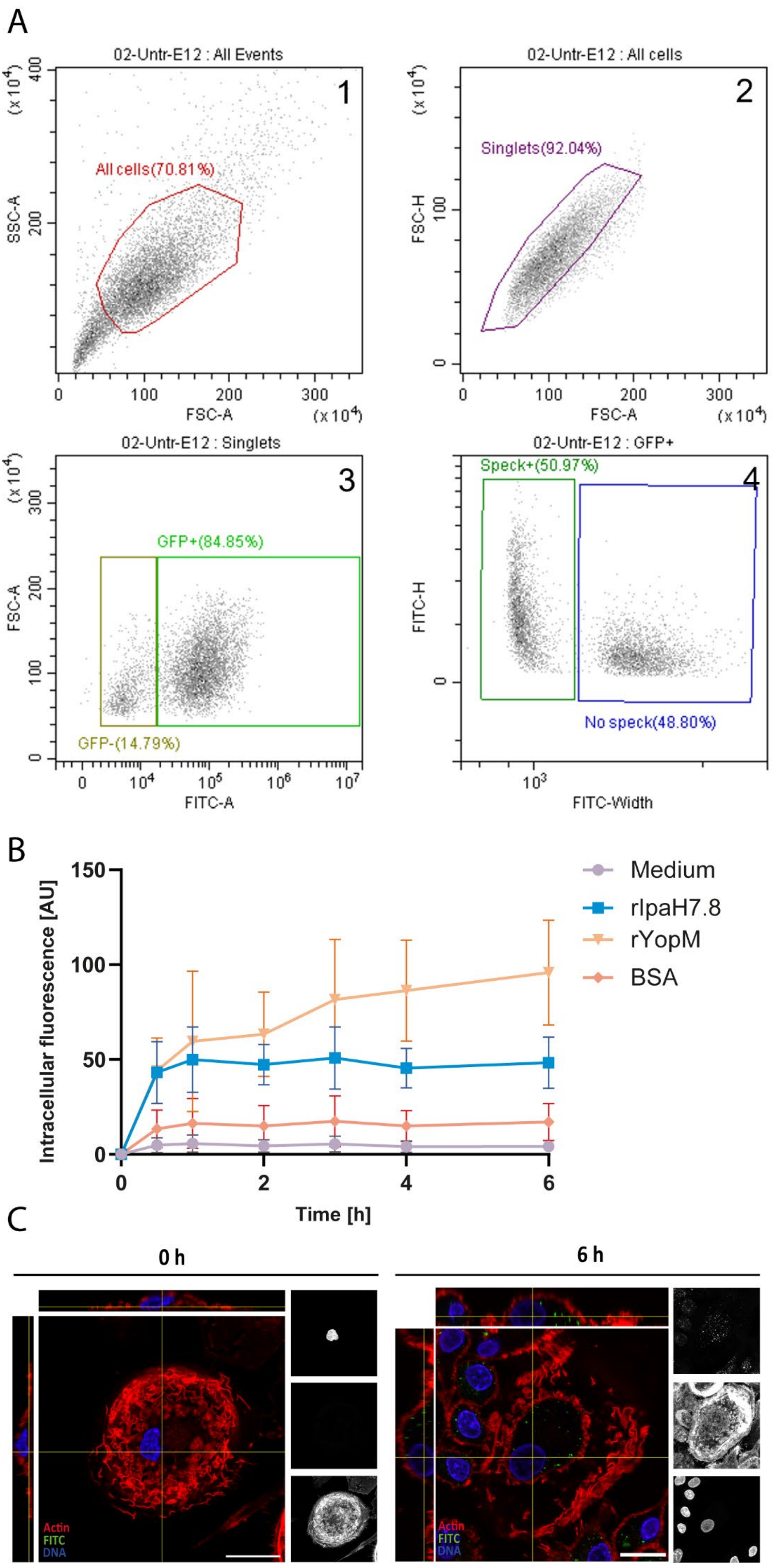

1564

bioRxiv preprint doi: https://doi.org/10.64898/2026.04.28.721327; this version posted May 1, 2026. The copyright holder for this preprint (which was not certified by peer review) is the author/funder, who has granted bioRxiv a license to display the preprint in perpetuity. It is made available under aCC-BY 4.0 International license.

Supplemental Figure S3. ASC speck gating strategy and uptake of rIpaH7.8 in primary human monocytes. (A) Flow cytometry gating strategy for ASC speck formation in THP-1 cells expressing ASC::GFP. Cells were gated for singlets and GFP-positive events, followed by discrimination of speck-positive and speck-negative populations based on FITC height versus width. (B) Uptake of FITC-labeled rIpaH7.8, rYopM, or BSA by primary human monocytes quantified by flow cytometry after incubation for up to 6 h. Medium-only served as a negative control. n = 7 donors; mean ± SD; AU, arbitrary units. (C) Confocal microscopy of primary human monocyte-derived macrophages incubated with FITC-labeled rIpaH7.8 (1.6 μM, 6 h). Actin (red), rIpaH7.8 (green), nuclei (blue). Medium-only (0 h) served as control. Scale bar, 20 μm. Orthogonal and maximum projections show single optical sections (1 airy unit).

bioRxiv preprint doi: https://doi.org/10.64898/2026.04.28.721327; this version posted May 1, 2026. The copyright holder for this preprint (which was not certified by peer review) is the author/funder, who has granted bioRxiv a license to display the preprint in perpetuity. It is made available under aCC-BY 4.0 International license.

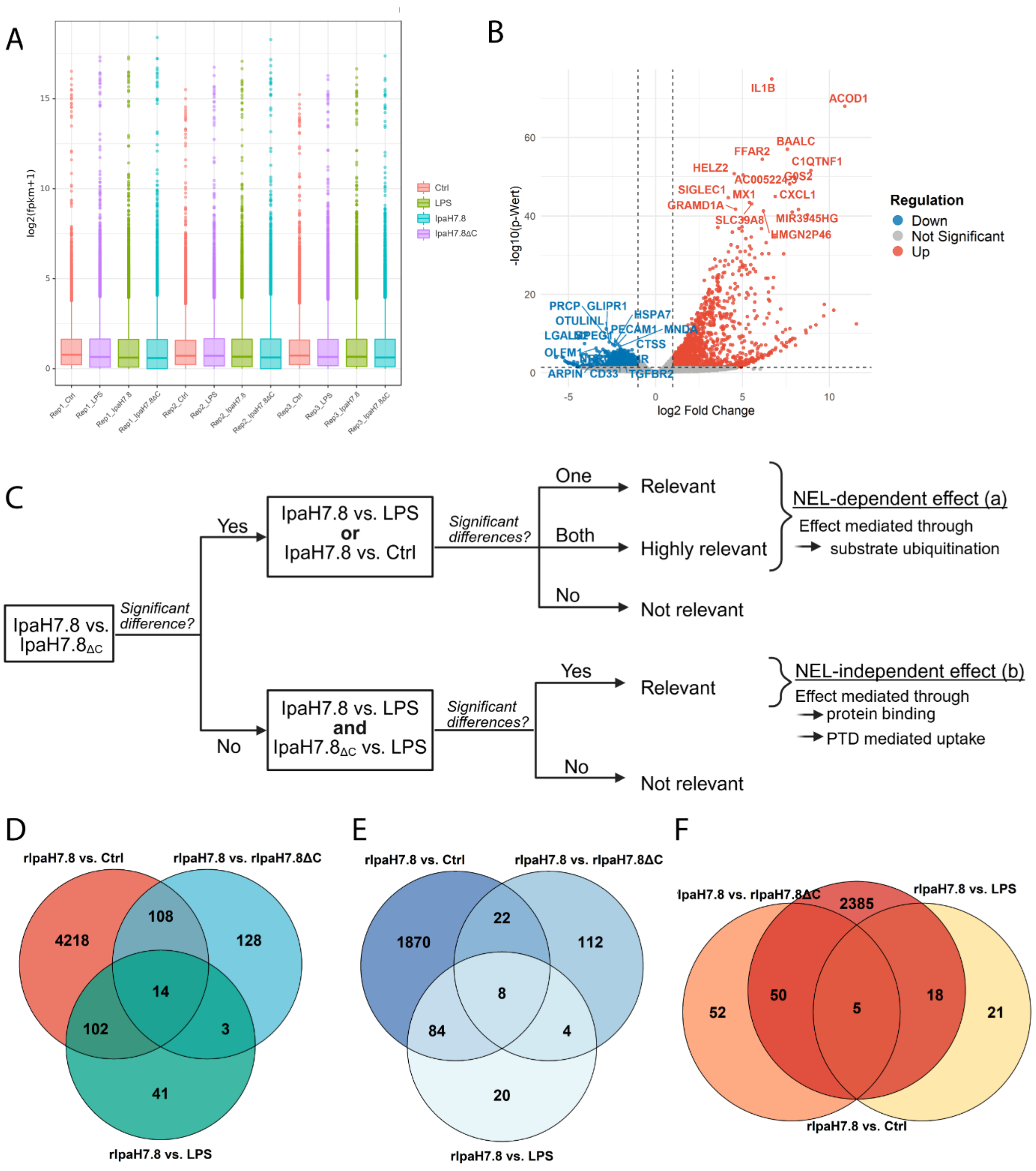

Supplementary Figure S4. Transcriptomic quality control, differential expression, and analysis strategy. (A) Gene expression distributions across biological replicates for each condition (Ctrl, LPS, LPS + rIpaH7.8, LPS + rIpaH7.8ΔC), demonstrating comparable global expression profiles. (B) Volcano plot showing differential gene expression in LPS-treated versus untreated monocytes. (C) Schematic overview of the pairwise comparison strategy used to define NEL-dependent and NEL-independent gene regulation. NEL-dependent genes were defined as differentially expressed exclusively in cells treated with catalytically active rIpaH7.8, whereas NEL-independent genes showed comparable regulation in both rIpaH7.8- and rIpaH7.8ΔC-treated cells ($p < 0.05$). For clarity, LPS co-treatment present in all conditions is not depicted. (D-F) Venn diagram analyses of differentially expressed genes across the indicated comparisons (Fig 4C-E), showing total (D), downregulated (E), and upregulated (F) DEGs.

bioRxiv preprint doi: https://doi.org/10.64898/2026.04.28.721327; this version posted May 1, 2026. The copyright holder for this preprint (which was not certified by peer review) is the author/funder, who has granted bioRxiv a license to display the preprint in perpetuity. It is made available under aCC-BY 4.0 International license.

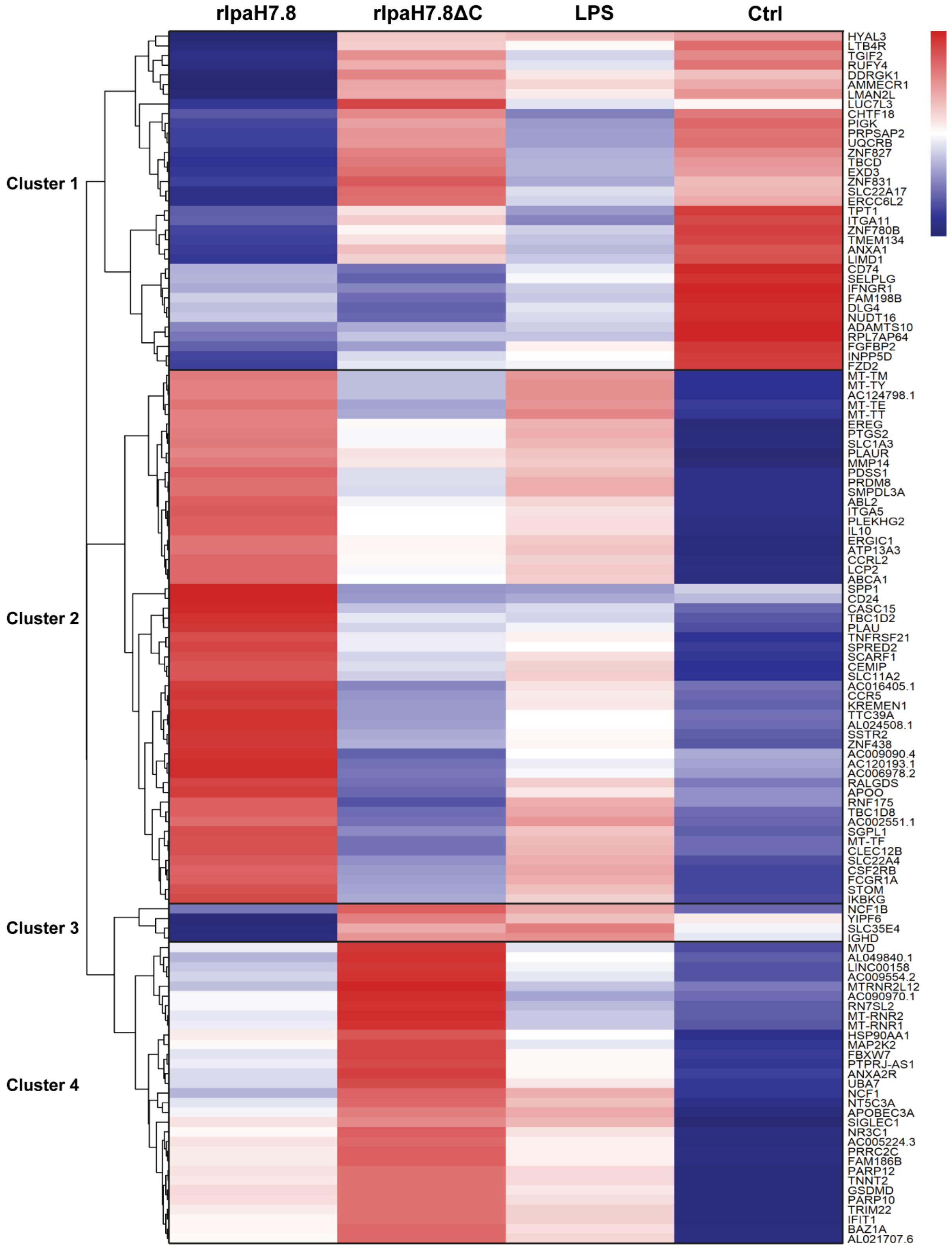

Supplementary Figure S5. rlpaH7.8-dependent transcriptional signatures in LPS-stimulated monocytes. Heatmap of differentially expressed genes (DEGs) identified by the intersectional filtering strategy shown in Fig. S4D and summarized in Fig. 4F. Expression values are shown as Z-score-normalized FPKM values per gene to emphasize relative differences across conditions. Rows represent individual genes clustered by Euclidean distance, and columns correspond to the indicated treatment conditions (LPS + rlpaH7.8, LPS + rlpaH7.8ΔC, LPS only, and untreated control). Red indicates higher relative expression and blue indicates lower relative expression. Unsupervised clustering indicates four major gene expression patterns.

bioRxiv preprint doi: https://doi.org/10.64898/2026.04.28.721327; this version posted May 1, 2026. The copyright holder for this preprint (which was not certified by peer review) is the author/funder, who has granted bioRxiv a license to display the preprint in perpetuity. It is made available under aCC-BY 4.0 International license.

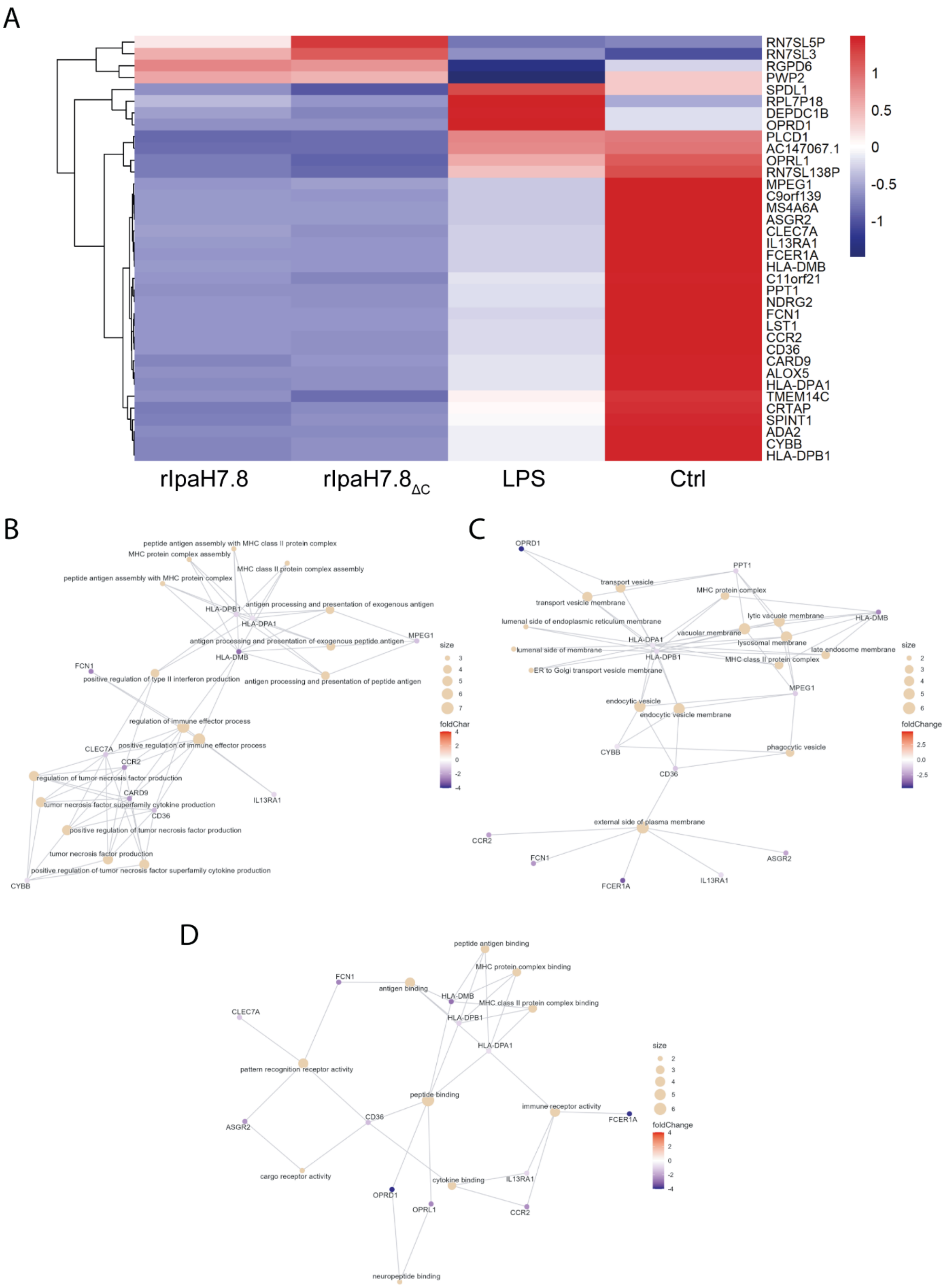

bioRxiv preprint doi: https://doi.org/10.64898/2026.04.28.721327; this version posted May 1, 2026. The copyright holder for this preprint (which was not certified by peer review) is the author/funder, who has granted bioRxiv a license to display the preprint in perpetuity. It is made available under aCC-BY 4.0 International license.

Supplemental Figure S6. Transcriptomic changes independent of NEL activity. (A) Heatmap of NEL-independent differentially expressed genes identified by the intersectional strategy shown in Fig. S4C. Z-score-normalized FPKM values are shown for each gene (rows), clustered by Euclidean distance, across the indicated conditions (rIpaH7.8, rIpaH7.8ΔC, LPS, and untreated). Red indicates higher and blue lower relative expression. (B-D) GO enrichment analysis of NEL-independent overlapping DEGs displayed as cnet plots for (B) Biological Processes, (C) Cellular Components, and (D) Molecular Functions. GO terms are shown as central nodes, with connected gene nodes indicating membership.

bioRxiv preprint doi: https://doi.org/10.64898/2026.04.28.721327; this version posted May 1, 2026. The copyright holder for this preprint (which was not certified by peer review) is the author/funder, who has granted bioRxiv a license to display the preprint in perpetuity. It is made available under aCC-BY 4.0 International license.

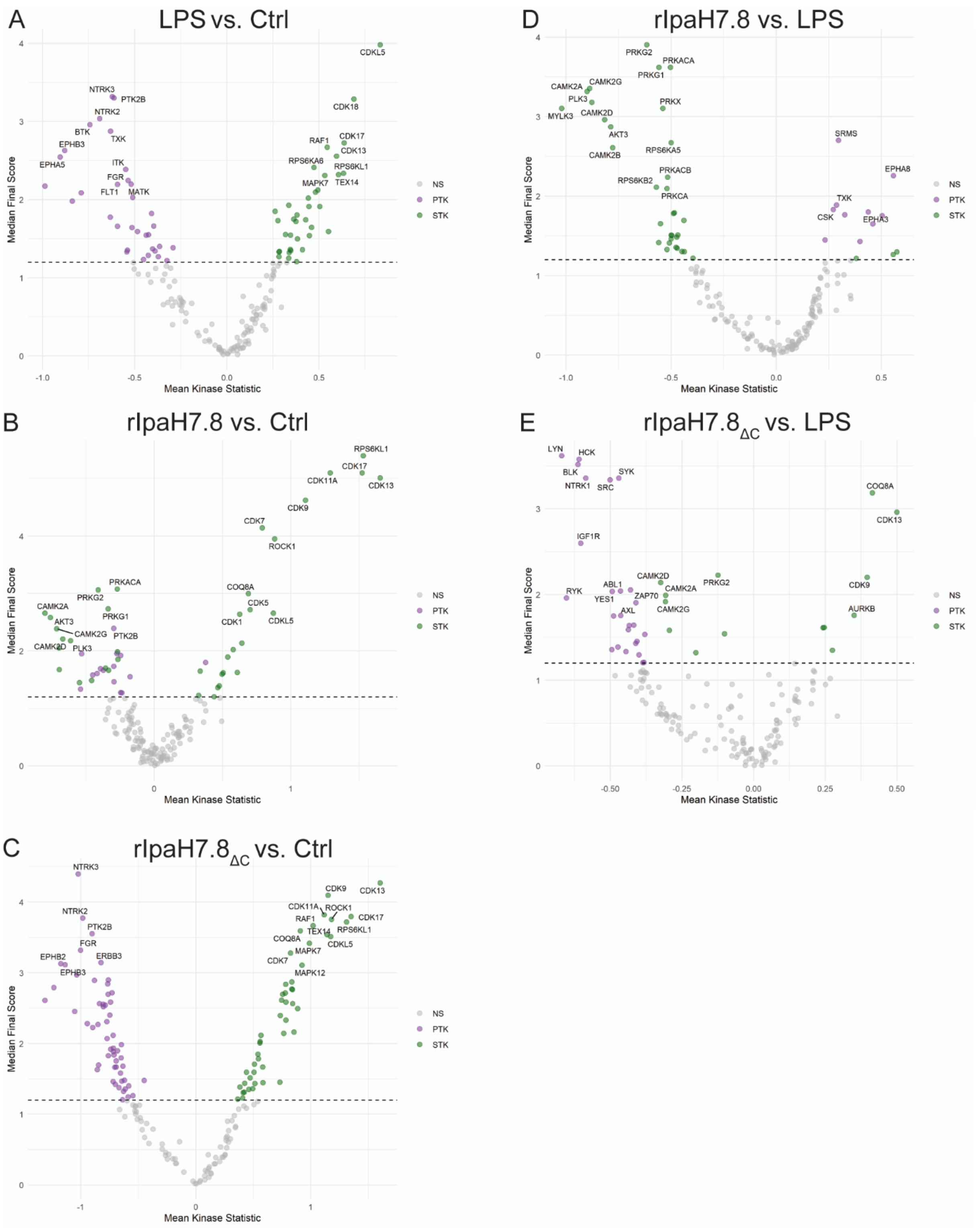

bioRxiv preprint doi: https://doi.org/10.64898/2026.04.28.721327; this version posted May 1, 2026. The copyright holder for this preprint (which was not certified by peer review) is the author/funder, who has granted bioRxiv a license to display the preprint in perpetuity. It is made available under aCC-BY 4.0 International license.

Supplemental Figure S7. Kinome profiling reveals rIpaH7.8-dependent modulation of LPS-induced kinase signaling. Primary human monocytes were analyzed using PamGene kinase arrays to infer upstream kinase activity changes under the indicated conditions. (A) Volcano plot showing inferred kinase activity changes following LPS stimulation compared to untreated control cells, revealing broad alterations in PTK and STK activities. (B) Kinase activity changes in monocytes treated with catalytically active rIpaH7.8 compared to control cells, indicating direct effector-mediated modulation of selected kinase families. (C) Kinase activity profile of cells treated with the catalytically inactive rIpaH7.8ΔC mutant compared to control, demonstrating partially overlapping but distinct, NEL-independent effects. (D) Comparison of LPS-stimulated cells treated with rIpaH7.8 versus LPS alone, showing partial reversal of LPS-induced kinase attenuation and restoration of specific immune-related kinase activities. (E) Comparison of LPS-stimulated cells treated with rIpaH7.8ΔC versus LPS alone, highlighting the requirement of the NEL domain for effective modulation of LPS-driven kinase responses. Each dot represents a kinase-associated peptide; significantly regulated peptides are highlighted (PTK, purple; STK, green), while non-significant peptides are shown in grey. The dashed line indicates the significance threshold (median final score > 1.2).

bioRxiv preprint doi: https://doi.org/10.64898/2026.04.28.721327; this version posted May 1, 2026. The copyright holder for this preprint (which was not certified by peer review) is the author/funder, who has granted bioRxiv a license to display the preprint in perpetuity. It is made available under aCC-BY 4.0 International license.

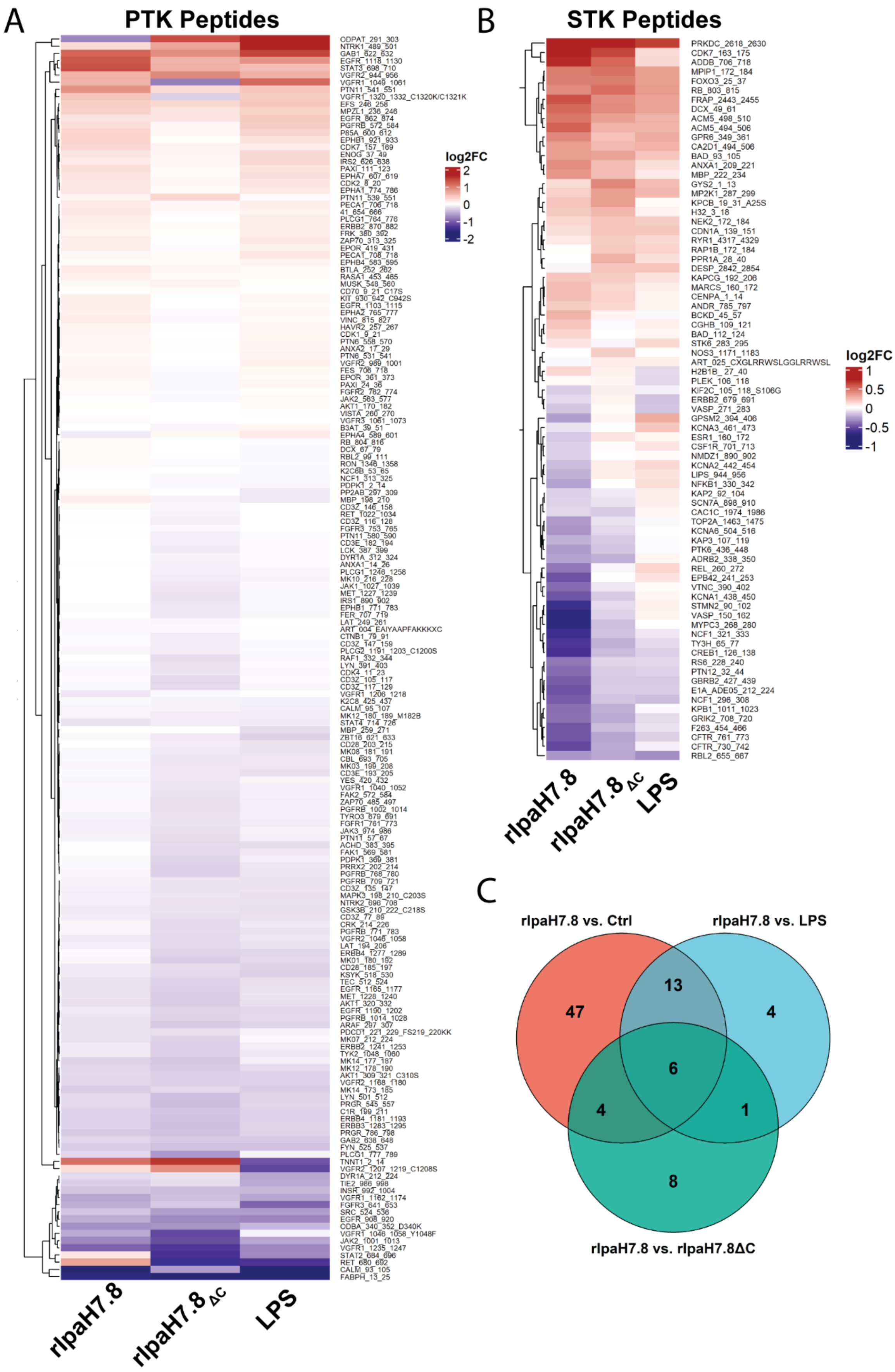

bioRxiv preprint doi: https://doi.org/10.64898/2026.04.28.721327; this version posted May 1, 2026. The copyright holder for this preprint (which was not certified by peer review) is the author/funder, who has granted bioRxiv a license to display the preprint in perpetuity. It is made available under aCC-BY 4.0 International license.

Supplemental Figure S8. Relative peptide phosphorylation profiles in response to rIpaH7.8. Primary human monocytes were stimulated with LPS (1 µg/mL) alone or in combination with rIpaH7.8 or rIpaH7.8ΔC (1.6 µM) for 6 h. Cell lysates were analyzed using PamGene kinase microarrays. Clustered heatmaps display log2 fold changes (log2FC) in phosphorylation of peptide substrates derived from protein tyrosine kinases (PTK, A) or serine/threonine kinases (STK, B) relative to untreated controls. Blue indicates reduced and red increased phosphorylation. Data represent three biological replicates, each consisting of pooled samples from three independent donors. (C) Venn diagram analyses of differentially phosphorylated peptides across the indicated comparisons.

bioRxiv preprint doi: https://doi.org/10.64898/2026.04.28.721327; this version posted May 1, 2026. The copyright holder for this preprint (which was not certified by peer review) is the author/funder, who has granted bioRxiv a license to display the preprint in perpetuity. It is made available under aCC-BY 4.0 International license.

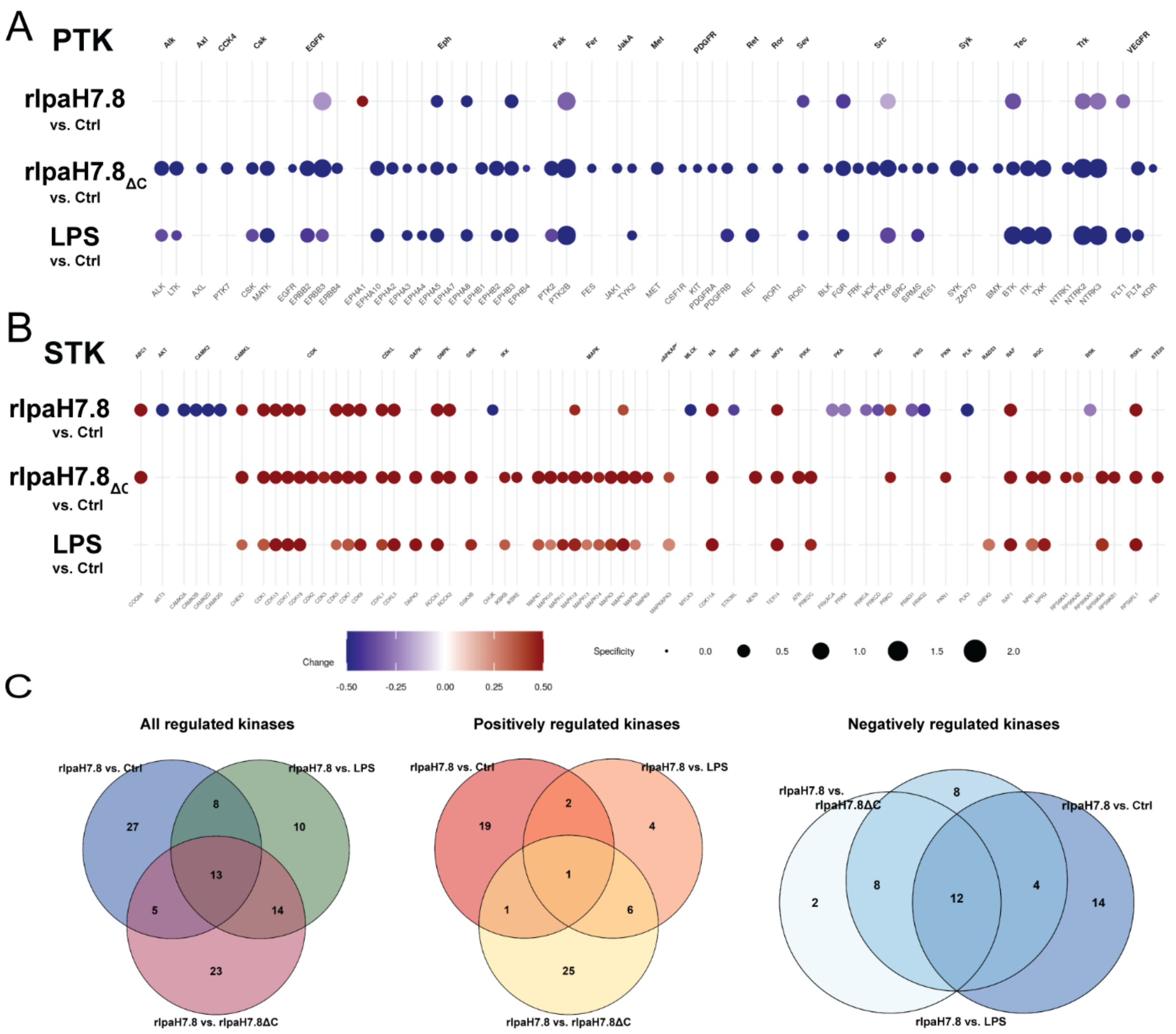

Supplemental Figure S9. Kinome profiling of rlpaH7.8-treated human monocytes. (A) PTK and STK (B) activity across conditions. Color indicates relative activity changes (blue, decreased; white, unchanged; red, increased); node size reflects substrate specificity. (C) Venn diagrams depicting kinases significantly affected by rlpaH7.8 across comparisons, including all, positively regulated, and negatively regulated kinases.

bioRxiv preprint doi: https://doi.org/10.64898/2026.04.28.721327; this version posted May 1, 2026. The copyright holder for this preprint (which was not certified by peer review) is the author/funder, who has granted bioRxiv a license to display the preprint in perpetuity. It is made available under aCC-BY 4.0 International license.

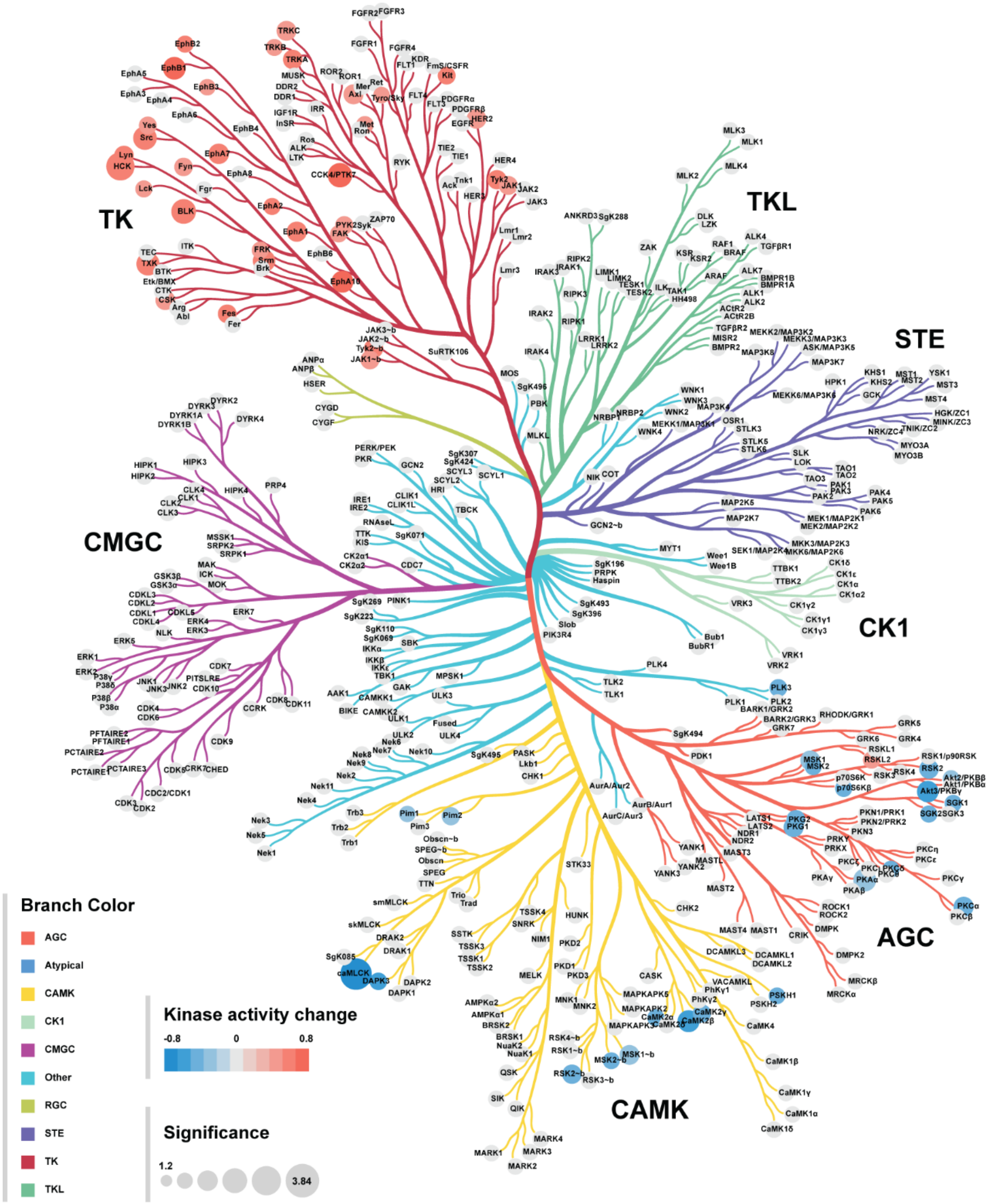

Supplemental Figure S10. Kinome tree visualization of rIpaH7.8-induced kinase modulation. Differentially active kinases identified by upstream kinase analysis were mapped onto the human kinome tree. Node size reflects predicted kinase activity scores, and node color indicates the direction of regulation in rIpaH7.8-treated cells relative to rIpaH7.8ΔC. Affected kinases cluster across multiple families, including AGC, CMGC, and TKL, indicating broad catalytic activity-dependent reprogramming of host kinase signaling.

bioRxiv preprint doi: https://doi.org/10.64898/2026.04.28.721327; this version posted May 1, 2026. The copyright holder for this preprint (which was not certified by peer review) is the author/funder, who has granted bioRxiv a license to display the preprint in perpetuity. It is made available under aCC-BY 4.0 International license.

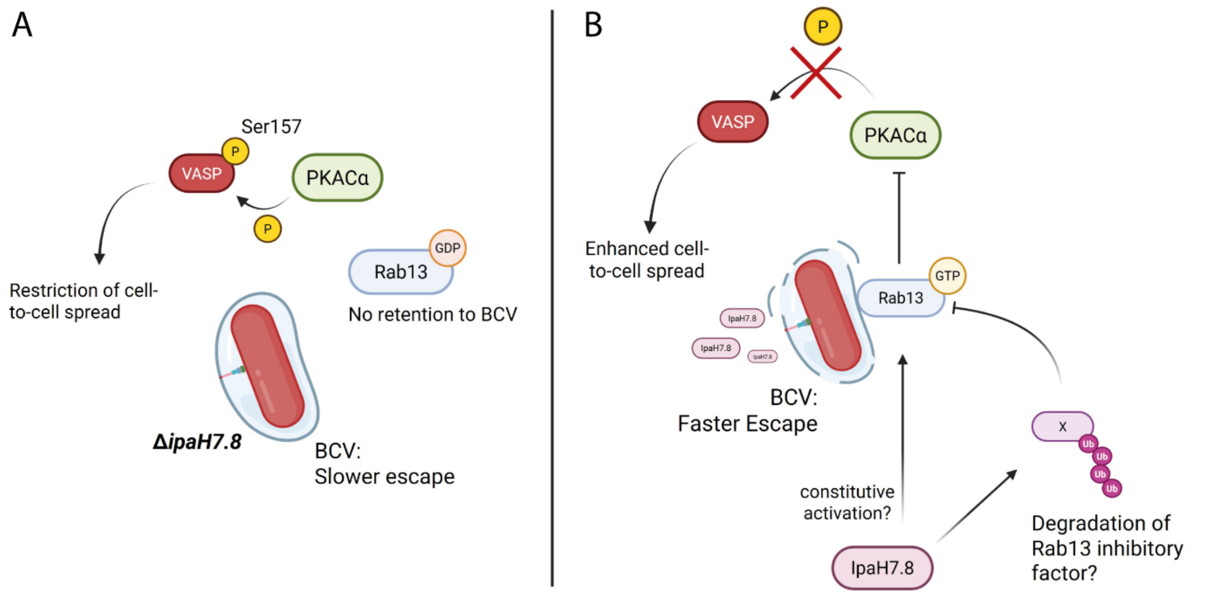

Supplemental Figure 11: Proposed mechanism of IpaH7.8 mediated promotion of vacuolar escape of *Shigella*. (A) In absence of IpaH7.8 mediated Rab13-retention, VASP Ser157 phosphorylation is conducted by PKACα, which in turn restricts cell-to-cell dissemination. (B) In presence of IpaH7.8, Rab13 is kept in its, active GTP bound form, leading to inhibition of PKACα and thus prevents VASP phosphorylation. This abolishes cell-to-cell spread restriction and in a so far unclear mechanism promotes vacuolar disassembly.

bioRxiv preprint doi: https://doi.org/10.64898/2026.04.28.721327; this version posted May 1, 2026. The copyright holder for this preprint (which was not certified by peer review) is the author/funder, who has granted bioRxiv a license to display the preprint in perpetuity. It is made available under aCC-BY 4.0 International license.

Supplemental Table 1: Numeric values of most regulated genes by rIpaH7.8 compared to LPS stimulation alone.

		IpaH7.8 vs Ctrl		IpaH7.8 vs LPS		IpaH7.8 vs IpaH7.8$_{ΔC}$		LPS vs. Ctrl	
Gene_ID	Name	log2FC	p-value	log2FC	p-value	log2FC	p-value	log2FC	p-value
ENSG00000272398	CD24	2.347	**0.019**	2.565	**0.021**	2.958	**0.022**	-0.180	0.887
ENSG00000118785	SPP1	1.535	**0.020**	1.966	**5.88E-03**	1.997	**0.022**	-0.389	0.537
ENSG00000272168	CASC15	3.660	**7.96E-07**	1.595	**0.013**	1.573	**0.042**	2.072	**0.031**
ENSG00000198369	SPRED2	2.896	**4.89E-11**	0.853	**0.043**	0.920	**0.038**	2.046	**9.89E-05**
ENSG00000095383	TBC1D2	1.132	**5.52E-04**	0.654	**0.038**	0.604	**0.047**	0.480	0.234
ENSG00000043462	LCP2	1.983	**7.42E-16**	0.330	0.218	0.577	**0.010**	1.661	**5.96E-07**
ENSG00000161638	ITGA5	1.487	**1.08E-09**	0.401	0.144	0.500	**0.041**	1.092	**3.96E-04**
ENSG00000135046	ANXA1	-0.987	**0.003**	-0.408	0.268	-0.773	**0.033**	-0.591	0.030
ENSG00000168918	INPP5D	-1.619	**1.17E-11**	-0.969	**0.003**	-0.817	**8.88E-03**	-0.661	**0.032**
ENSG00000158517	NCF1	0.723	**0.001**	-0.580	**0.020**	-0.819	**8.31E-05**	1.309	**3.66E-06**
ENSG00000182487	NCF1B	0.131	0.721	-0.769	**0.041**	-0.977	**0.010**	0.900	**0.004**
ENSG00000211898	IGHD	-0.861	**0.075**	-1.333	**8.70E-03**	-1.304	**0.038**	0.477	0.253
ENSG00000181704	YIPF6	-1.197	**0.038**	-1.388	**2.29E-02**	-1.582	**0.020**	0.189	0.678
ENSG00000114988	LMAN2L	-1.781	**5.58E-03**	-1.520	**0.040**	-1.764	**0.017**	-0.276	0.599
ENSG00000188282	RUFY4	-2.003	**1.46E-04**	-1.412	**0.035**	-1.905	**0.008**	-0.606	0.219
ENSG00000180340	FZD2	-3.031	**2.30E-06**	-2.063	**0.013**	-1.944	**0.032**	-0.981	0.056
ENSG00000128000	ZNF780B	-3.144	**1.07E-06**	-1.872	**0.038**	-2.246	**0.014**	-1.273	**0.017**
ENSG00000100036	SLC35E4	-2.320	**0.083**	-3.006	**0.021**	-2.838	**0.045**	0.689	0.379
ENSG00000137809	ITGA11	-4.001	**2.56E-03**	-1.257	0.562	-3.282	**0.040**	-2.811	**7.43E-03**
ENSG00000186792	HYAL3	-4.783	**4.21E-03**	-4.666	**0.015**	-4.604	**0.022**	-0.180	0.851
ENSG00000213876	RPL7AP64	-7.319	**1.79E-08**	-5.037	**5.76E-03**	-4.925	**0.023**	-2.366	**7.76E-04**

Supplemental Table S2: Contrasts of peptide phosphorylation analysis

(attached as supplemental file)

bioRxiv preprint doi: https://doi.org/10.64898/2026.04.28.721327; this version posted May 1, 2026. The copyright holder for this preprint (which was not certified by peer review) is the author/funder, who has granted bioRxiv a license to display the preprint in perpetuity. It is made available under aCC-BY 4.0 International license.

Supplemental Table 3: List of relevant kinases significantly regulated by rIpaH7.8 treatment (NEL dependent; IpaH7.8 vs. IpaH7.8 $_{\Delta C}$).

Kinase	Uniprot-ID	Median Final score (Specificity)	Mean Kinase Statistic (Kinase activity change)	Chip
MYLK3	Q32MK0	3.854	-0.874	STK
PTK7	Q13308	2.601	0.719	PTK
TXK	P42681	2.387	0.546	PTK
PRKACA	P17612	2.322	-0.356	STK
SRMS	Q9H3Y6	2.255	0.532	PTK
CAMK2B	Q13554	2.179	-0.582	STK
EPHA10	Q5JZY3	2.158	0.727	PTK
AKT3	Q9Y243	2.088	-0.559	STK
EPHA1	P21709	1.863	0.598	PTK
PRKCA	P17252	1.834	-0.412	STK
PRKG1	Q13976	1.834	-0.368	STK
RPS6KA5	O75582	1.793	-0.344	STK
CSK	P41240	1.785	0.488	PTK
RPS6KA3	P51812	1.724	-0.411	STK
PRKG2	Q13237	1.631	-0.367	STK
FES	P07332	1.608	0.539	PTK
CAMK2G	Q13555	1.525	-0.453	STK
PIM2	Q9P1W9	1.512	-0.342	STK
PLK3	Q9H4B4	1.427	-0.423	STK
RPS6KL1	Q9Y6S9	1.379	0.488	STK
CAMK2A	Q9UQM7	1.340	-0.465	STK
RPS6KB2	Q9UBS0	1.333	-0.378	STK
SGK2	Q9HBY8	1.332	-0.415	STK
SGK1	O00141	1.324	-0.405	STK
PRKCD	Q05655	1.299	-0.372	STK
RPS6KA4	O75676	1.285	-0.437	STK
PSKH1	P11801	1.239	-0.478	STK
AKT2	P31751	1.231	-0.353	STK

bioRxiv preprint doi: https://doi.org/10.64898/2026.04.28.721327; this version posted May 1, 2026. The copyright holder for this preprint (which was not certified by peer review) is the author/funder, who has granted bioRxiv a license to display the preprint in perpetuity. It is made available under aCC-BY 4.0 International license.

Supplemental Table 4: List of relevant kinases significantly regulated by rIpaH7.8 treatment (NEL independent; IpaH7.8 vs. LPS).
Full list of peptide regulation can be accessed at xxxDOI; Suppl_Data_S4 Tab

Kinase	Uniprot-ID	Median Final score (Specificity)	Mean Kinase Statistic (Kinase activity change)	Chip
PRKX	P51817	3.106	-0.269	STK
CAMK2D	Q13557	2.963	-0.671	STK
CDK13	Q14004	1.265	1.655	STK
